# *Escherichia coli* Can Adapt Its Protein Translocation Machinery for Enhanced Periplasmic Recombinant Protein Production

**DOI:** 10.3389/fbioe.2019.00465

**Published:** 2020-01-29

**Authors:** Alexandros Karyolaimos, Katarzyna Magdalena Dolata, Minia Antelo-Varela, Anna Mestre Borras, Rageia Elfageih, Susanne Sievers, Dörte Becher, Katharina Riedel, Jan-Willem de Gier

**Affiliations:** ^1^Department of Biochemistry and Biophysics, Center for Biomembrane Research, Stockholm University, Stockholm, Sweden; ^2^Institute of Microbiology, University of Greifswald, Greifswald, Germany

**Keywords:** *Escherichia coli*, recombinant protein, protein production, periplasm, Sec-translocon, LepB, YidC

## Abstract

Recently, we engineered a tunable rhamnose promoter-based setup for the production of recombinant proteins in *E. coli*. This setup enabled us to show that being able to precisely set the production rate of a secretory recombinant protein is critical to enhance protein production yields in the periplasm. It is assumed that precisely setting the production rate of a secretory recombinant protein is required to harmonize its production rate with the protein translocation capacity of the cell. Here, using proteome analysis we show that enhancing periplasmic production of human Growth Hormone (hGH) using the tunable rhamnose promoter-based setup is accompanied by increased accumulation levels of at least three key players in protein translocation; the peripheral motor of the Sec-translocon (SecA), leader peptidase (LepB), and the cytoplasmic membrane protein integrase/chaperone (YidC). Thus, enhancing periplasmic hGH production leads to increased Sec-translocon capacity, increased capacity to cleave signal peptides from secretory proteins and an increased capacity of an alternative membrane protein biogenesis pathway, which frees up Sec-translocon capacity for protein secretion. When cells with enhanced periplasmic hGH production yields were harvested and subsequently cultured in the absence of inducer, SecA, LepB, and YidC levels went down again. This indicates that when using the tunable rhamnose-promoter system to enhance the production of a protein in the periplasm, *E. coli* can adapt its protein translocation machinery for enhanced recombinant protein production in the periplasm.

## Introduction

The bacterium *Escherichia coli* is widely used for the production of recombinant proteins (Rosano et al., 2019). Disulfide bond containing recombinant proteins are usually produced in the periplasm (Bardwell et al., 1991; Baneyx and Mujacic, 2004; Landeta et al., 2018). In the periplasm, the oxidizing environment and the disulfide bond formation (Dsb)-system facilitate the formation of disulfide bonds (Bardwell et al., 1991; Baneyx and Mujacic, 2004; Landeta et al., 2018). However, the bottlenecks associated with guiding recombinant proteins across the cytoplasmic membrane can negatively affect protein production yields in the periplasm (Baneyx and Mujacic, 2004; De Geyter et al., 2016).

To target a recombinant protein to the periplasm, it is usually fused at the N-terminus to a signal peptide guiding it to the Sec-translocon (Crane and Randall, 2017; Tsirigotaki et al., 2017). The Sec-translocon not only mediates the translocation of secretory proteins across the cytoplasmic membrane but also the insertion of membrane proteins into the cytoplasmic membrane (Crane and Randall, 2017; Tsirigotaki et al., 2017). The core of the Sec-translocon consists of SecY and SecE, which are integral membrane proteins and together they form a protein-conducting channel in the cytoplasmic membrane, as well as the peripheral ATP-dependent motor protein SecA, which pushes secretory proteins and sizable periplasmic domains of cytoplasmic membrane proteins through the Sec-translocon channel (Crane and Randall, 2017; Tsirigotaki et al., 2017). During translocation across the cytoplasmic membrane, secretory proteins are mostly in an unfolded conformation (Arkowitz et al., 1993). Upon translocation of a protein leader peptidase (LepB) cleaves off the signal peptide, and the protein subsequently folds in the periplasm (Paetzel, 2014; Tsirigotaki et al., 2017). The cytoplasmic membrane protein integrase/chaperone YidC can assist the biogenesis of cytoplasmic membrane proteins in conjunction with the Sec-translocon as well as an independent entity (Kuhn et al., 2017).

It is assumed that in order to enhance the periplasmic production yield of a recombinant protein, the production rate of the precursor form of the protein should be harmonized with the capacity of the Sec-translocon (Schlegel et al., 2013; Horga et al., 2018). Saturation of the Sec-translocon capacity can have negative effects on the formation of biomass and the production of proteins in the periplasm (Schlegel et al., 2013; Hjelm et al., 2017; Baumgarten et al., 2018; Horga et al., 2018). To harmonize the production rate of a secretory recombinant protein with the Sec-translocon capacity, a tunable protein production system should be used. Recently, by combining a rhamnose promoter-based expression vector and a strain background deficient in the rhamnose operon, a setup was created that enables precise regulation of protein production rates by varying the amount of rhamnose in the culture medium (Hjelm et al., 2017; Karyolaimos et al., 2019).

This experimental system recently allowed us to enhance the periplasmic production of the disulfide bond containing recombinant protein human Growth Hormone (hGH) (Karyolaimos et al., 2019). Here, by analyzing the proteome composition of cells with enhanced periplasmic hGH production yields, we were able to show that the protein translocation machinery of *E. coli* can adapt for enhanced periplasmic recombinant protein production.

## Materials and Methods

### Strain, Expression Vectors, and hGH Production Experiments

**Figure 1:**
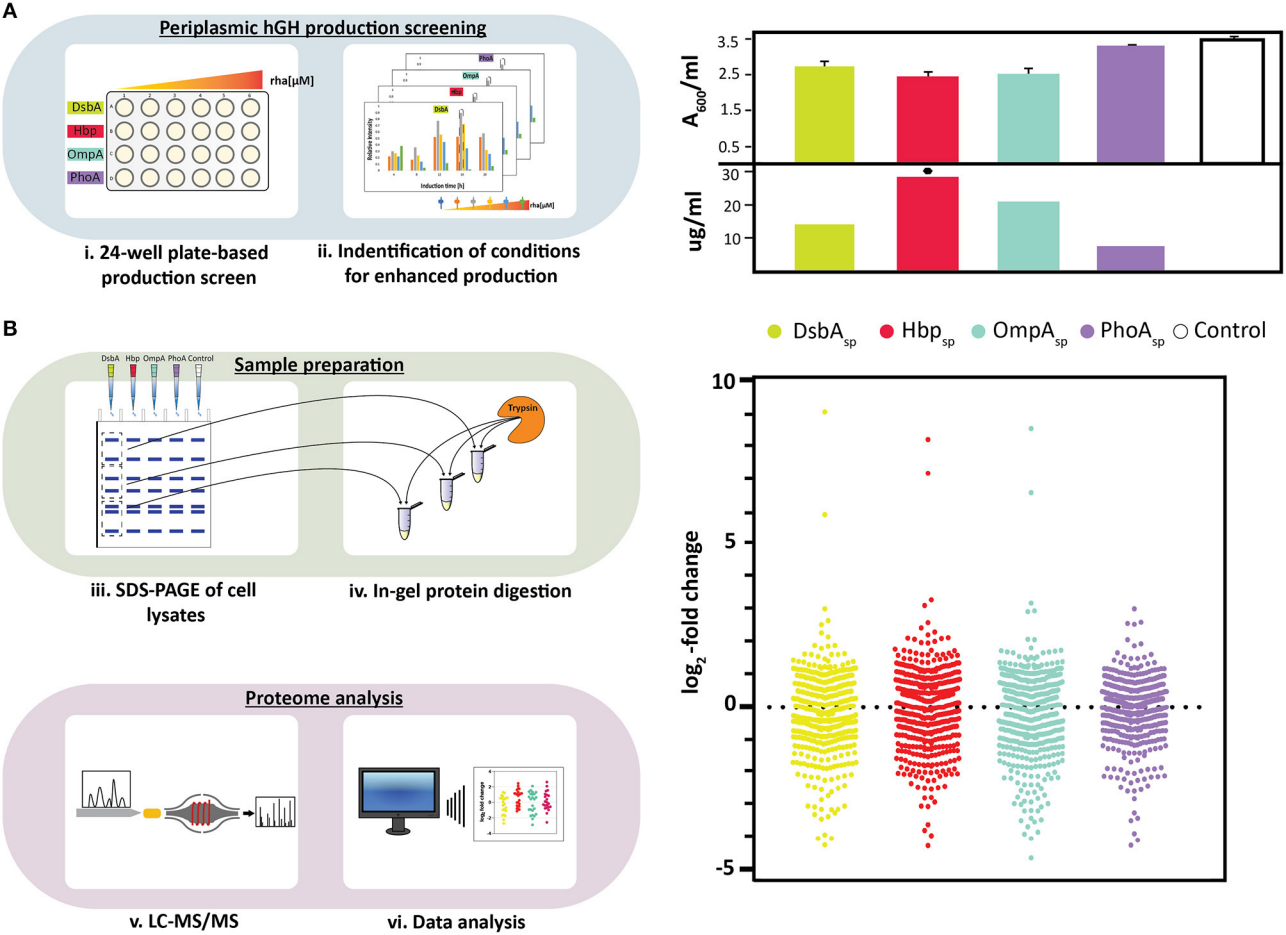
Periplasmic hGH production screening and proteome analysis of hGH producing cells. **(A)** On the left: Periplasmic production screening of hGH fused to different signal peptides (DsbA_sp_, Hbp_sp_, OmpA_sp_, and PhoA_sp_) was done in standard 24-well plates (i). Expression of the *hgh* gene fused to the genetic information encoding the four different signal peptides was induced using varying amounts of rhamnose. The production of hGH in the periplasm was monitored over time by separating proteins present in equal volumes of culture by SDS-PAGE followed by immuno-blotting using a fluorescently labeled antibody recognizing the C-terminal His_6_-tag of hGH. This enabled to make for each of the signal peptides used a relative comparison of the hGH production yields in the periplasm (ii). On the right: For each signal peptide used, both biomass formation (A_600_, based on three independent biological replicates) and periplasmic hGH production (μg of hGH produced per ml of culture) were determined for the setup leading to the highest periplasmic hGH production yield per culture volume (see Materials and Methods). The highest volumetric periplasmic hGH production yield is indicated with a black hexagon. **(B)** On the left: Proteins in whole cell lysates were separated by SDS-PAGE (iii). A whole cell lysate derived of cells with an empty expression vector, i.e., not producing any hGH, were used as a reference. Proteins present in different sections of the gel were digested using trypsin (iv) and the digested material was subsequently analyzed by LC-MS/MS (v). Relative quantification of identified proteins was done using the label free quantification (LFQ) algorithm (vi). On the right: Relative abundance of proteins in whole cell lysates derived of cells producing hGH in the periplasm employing DsbA_sp_, Hbp_sp_, OmpA_sp_, and PhoA_sp_ using whole cell lysates derived of cells with an empty expression vector as a reference. Distribution of changes in the relative abundance of proteins are shown in a vertical scatter plot; log_2_-fold changes that are statistically significant (*p* < 0.01, ANOVA, Supplementary Table 1) were plotted using GraphPad Prism as a Column graph.

The W3110Δ*rha* strain and rhamnose promoter-based expression vectors pRha-*dsbA*_*sp*_*hghhis*_6_, pRha-*hbp*_*sp*_*hghhis*_6_, pRha-*ompA*_*sp*_*hghhis*_6_, pRha-*phoA*_*sp*_*hghhis*_6_, and the empty pRha expression vector used in this study have been described previously (Karyolaimos et al., 2019). Cells were transformed with the aforementioned pRha expression vectors as well as an empty pRha expression vector that served as a control. Protein production was done in a standard 24-well plate format (culture volume 4 ml). Cells were grown aerobically at 30°C and 200 rpm (New Brunswick Innova 42R shaker with an orbit diameter of 1.9 cm), in Lysogeny Broth (LB; a.k.a. LB medium) supplemented with 50 μg/ml kanamycin. 0.2% glucose was added to the precultures to prevent any background expression of the genes encoding the target proteins. Growth was monitored by measuring the A_600_ with a UV-1300 spectrophotometer (Shimadzu). At an A_600_ of ~0.5 target gene expression was induced by the addition of rhamnose at a concentration optimal for the periplasmic production of hGH; for DsbA_sp_ 100 μM rhamnose, for Hbp_sp_ 50 μM rhamnose, for OmpA_sp_ 50 μM rhamnose, for PhoA_sp_ 100 μM rhamnose, and for the control 100 μM rhamnose was used (Karyolaimos et al., 2019). Cells were harvested 16 h after the addition of rhamnose for further characterization. Standard deviations shown in Figure 1A with the A_600_ measurements 16 h after the addition of rhamnose are based on at least three independent biological replicates. When hGH producing cells (as well as control cells with an empty expression vector) were used for another hGH production run, cells were harvested 16 h after the addition of rhamnose, washed three times with plain LB medium and the washed cells were subsequently used to inoculate a fresh culture with a starting A_600_ of 0.07.

### SDS-PAGE and Immuno-Blotting

To monitor the production of periplasmic hGH, whole-cell lysates were separated by sodium dodecyl sulfate-polyacrylamide gel electrophoresis (SDS-PAGE) followed by immuno-blotting using an AlexaFluor 647 conjugated anti-His antibody (Invitrogen) as described previously (Karyolaimos et al., 2019). Known amounts of isolated hGH were used for calibration (Karyolaimos et al., 2019). SecA, LepB, and YidC levels in whole cell lysates were monitored using polyclonal α-SecA, α-LepB, and α-YidC antisera from the lab collection (raised in rabbits) combined with a secondary Alexa Fluor 647 fluorescent conjugated goat-α-rabbit antibody (Invitrogen).

### Proteome Analysis of hGH Producing Cells

Protein digestion, liquid chromatography–tandem mass spectrometry (LC–MS/MS) analysis, protein identification and quantification methodology used to analyze the proteome of cells producing periplasmic hGH using different signal peptides and control cells with an empty expression vector have been described in detail previously (Guerrero Montero et al., 2019). In short, proteins in whole cell lysates were separated by SDS-PAGE and subsequently each lane was cut into 10 equidistant pieces prior to in-gel digestion using trypsin. The generated peptides were eluted from the gel to an aqueous solution by ultrasonication and the concentrated peptide mixture was subjected to LC-MS/MS analysis performed on an EASY-nLC II coupled to an LTQ Orbitrap (Thermo Fisher Scientific) mass spectrometer. Peptides were separated on in-house self-packed columns (Guerrero Montero et al., 2019) using a 100 min non-linear gradient from 1% acetonitrile and 0.1% acetic acid to 99% acetonitrile and 0.1% acetic acid at a flow rate of 300 nL/min. Precursor measurements were acquired in the Orbitrap (30,000 resolution) and the five most abundant precursor ions were selected for collision-induced dissociation fragmentation (CID). The mass spectrometry proteomics data have been deposited to the ProteomeXchange Consortium (http://proteomecentral.proteomexchange.org) *via* the PRIDE partner repository (Vizcaino et al., 2013) with the dataset identifier PXD013168 (username: reviewer89655@ebi.ac.uk, password: myvMo3bU). Database searching was done against a randomized *E. coli* K12 W3110 UniProt/Swissprot database (Proteome ID: UP000000318, 4257 entries, version December 2017) with the added amino acid sequence of hGH, using the MaxQuant software (version 1.5.8.3) (Cox and Mann, 2008). Proteins had to be identified in at least two out of 3 biological replicates, and a minimum of two unique peptides per protein was required for relative quantification using the label free quantification (LFQ) algorithm from MaxQuant (Cox et al., 2014). Mean values were calculated based on 3 biological replicates and relative protein abundance was represented as log_2_-fold change. ANOVA multivariance test was used to determine statistical significance of protein abundance and for each protein identified this is represented by a p value (*p* < 0.01).

## Results

### Consequences of Enhancing the Periplasmic Production of hGH

Recently, we used rhamnose promoter-based production rate screening in combination with four signal peptides, DsbA_sp_, Hbp_sp_, OmpA_sp_, and PhoA_sp_, to identify conditions for the enhanced production of hGH in the periplasm (Figure 1A, panel on the left) (Karyolaimos et al., 2019). Enhancing the periplasmic production of hGH had a minor negative effect on biomass formation when DsbA_sp_, Hbp_sp_, and OmpA_sp_ were used, and there was no significant effect on biomass formation when PhoA_sp_ was used (Figure 1A, panel on the right) (Karyolaimos et al., 2019). Periplasmic production of hGH was monitored by means of quantitative immuno-blotting using equal amounts of culture volume (Figure 1A, panel on the right) (Karyolaimos et al., 2019). Periplasmic hGH production was best when using the Hbp_sp_ and 50 μM of rhamnose, and resulted in properly folded and active hGH (Karyolaimos et al., 2019). With the DsbA_sp_ (100 μM rhamnose) and OmpA_sp_ (50 μM rhamnose), hGH was also efficiently produced in the periplasm, but yields were lower than with Hbp_sp_. Enhancing periplasmic hGH production with PhoA_sp_ (100 μM rhamnose) resulted in only a minute amount of hGH in the periplasm and most of the hGH was retained as the precursor protein in the cytoplasm in aggregates. Thus, different signal peptides can have a significant impact on the periplasmic production of hGH in the periplasm.

To gain more insight into the consequences of enhancing the periplasmic production of hGH, we analyzed the proteome composition of cells producing hGH using the aforementioned four signal peptides and the optimal rhamnose concentration (Figure 1B, top panel on the left). Cells with an empty expression vector, i.e., not producing hGH, were used as a reference. Proteins in whole cell lysates were separated by SDS-PAGE and subsequently the proteins present in different sections of the gel were identified by LC-MS/MS (Figure 1B, bottom panel on the left). Relative quantification of identified proteins was done using the LFQ algorithm (Cox and Mann, 2008) and significant changes in protein abundance were determined (Figure 1B, bottom panel on the left and panel on the right as well as Supplementary Table 1). This analysis indicated that enhanced periplasmic hGH production had a significant impact on the proteome composition of the producing cells and that using PhoA_sp_, which leads to the lowest periplasmic hGH production, led compared to using the other three signal peptides to a less dramatic overall change in the proteome composition. Out of the 509 proteins showing statistically significant changes, for the DsbA_sp_ Hbp_sp_, and OmpA_sp_ more than 250 were considerably changed (Figure 1B, Supplementary Table 1, proteins with *p* < 0.01 and log_2_-fold changes > 0.8, < −0.8, ON and OFF). However, for the PhoA_sp_ only about 150 proteins were considerably changed (Figure 1B, Supplementary Table 1).

### Enhancing hGH Production in the Periplasm Leads to Increased Accumulation Levels of SecA, LepB, and YidC

Since the rationale of using the rhamnose promoter-based setup to enhance periplasmic recombinant protein production is to harmonize secretory recombinant protein production rates with the capacity of the Sec-translocon, we expected that the accumulation levels of components involved in protein translocation would not be affected upon enhanced periplasmic hGH production (Schlegel et al., 2013; Horga et al., 2018).

**Figure 2:**
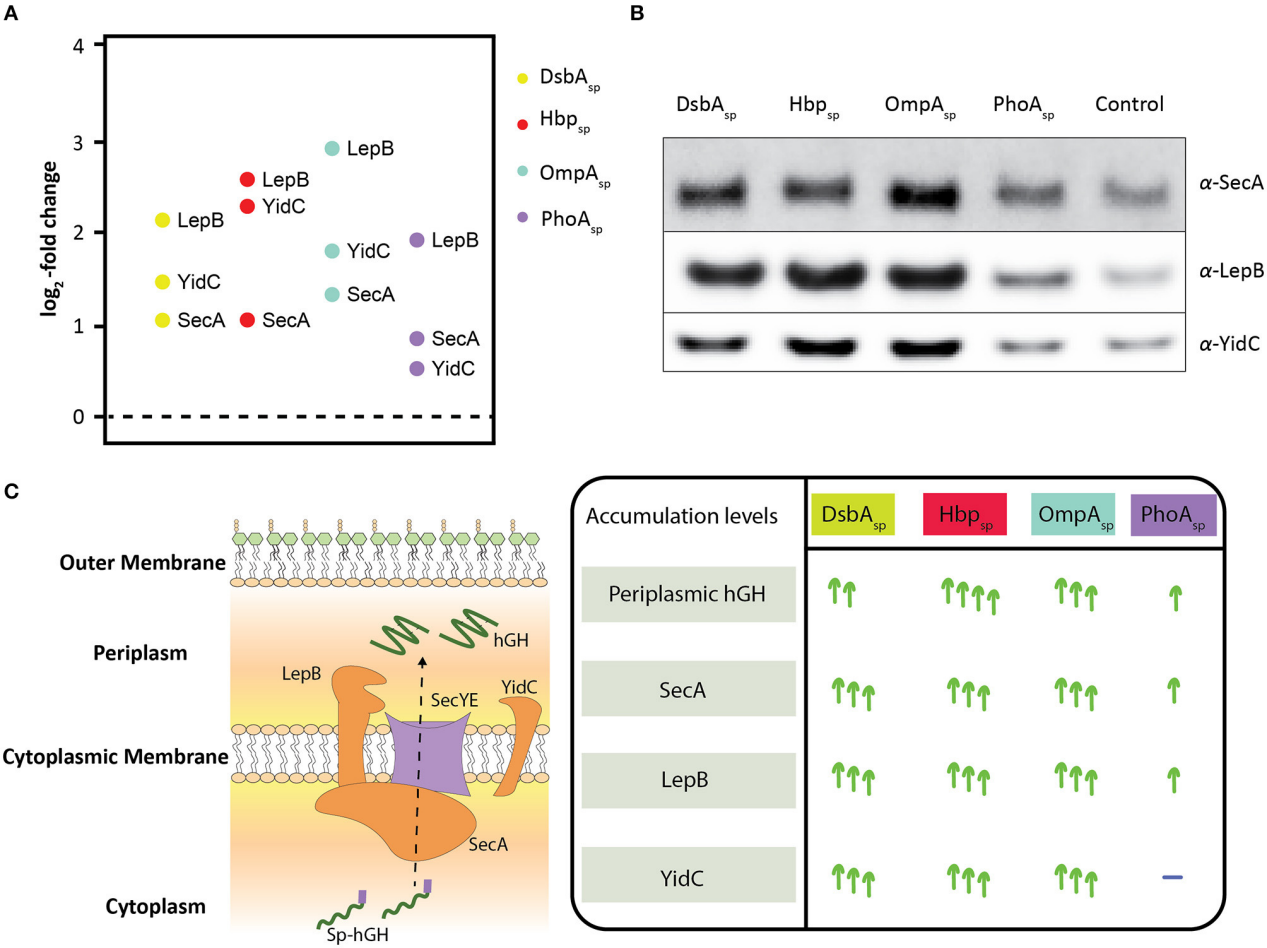
Effects of enhancing periplasmic hGH production using DsbA_sp_, Hbp_sp_, OmpA_sp_, and PhoA_sp_ on the accumulation levels of SecA, Lep, and YidC. **(A)** Three key components involved in protein translocation, SecA, LepB, and YidC, were identified showing statistically significant changes (Supplementary Table 1). The relative accumulation levels of SecA, LepB, and YidC were determined in cells producing hGH using DsbA_sp_, Hbp_sp_, OmpA_sp_, and PhoA_sp_. **(B)** Accumulation levels of SecA, LepB, and YidC were also monitored by SDS-PAGE/immuno-blotting by loading equal amounts of biomass and using antisera against SecA, LepB, and YidC. A secondary fluorescently labeled antibody was used for visualization. **(C)** On the left: Schematic representation of how secretory proteins are translocated to the periplasm *via* the Sec-translocon. The components involved in protein translocation identified and affected, SecA, LepB, and YidC, are in orange. On the right: Summary of how SecA, LepB, and YidC accumulation levels are affected upon the enhanced periplasmic hGH production using DsbA_sp_, Hbp_sp_, OmpA_sp_, and PhoA_sp_.

From the aforementioned key components involved in protein translocation SecY, SecE, SecA, LepB, and YidC, for only the last three components statistically significant changes in their accumulation levels were observed (Figure 2A, Supplementary Table 1). SecA is the peripheral ATP-dependent motor of the Sec-translocon (Tsirigotaki et al., 2017). LepB cleaves off signal peptides from secretory proteins upon their translocation across the cytoplasmic membrane (Tsirigotaki et al., 2017). The cytoplasmic membrane protein integrase/chaperone YidC in association with the Sec-translocon as well as an independent entity assists the biogenesis of membrane proteins (Welte et al., 2012). In contrast to what was expected, the relative accumulation levels of all these three components were up when DsbA_sp_, Hbp_sp_, and OmpA_sp_ were used (Figure 2A). When PhoA_sp_ was used, the accumulation levels of SecA and LepB were up, although not as much as for the other signal peptides, and the YidC accumulation level was not affected. Notably, periplasmic hGH production was lowest when PhoA_sp_ was used compared to the other three signal peptides (Figure 1A, panel on the right). These observations were corroborated by means of immuno-blotting (Figure 2B). The increased accumulation levels of SecA indicate that cells have a higher Sec-translocon capacity; it has been shown that when cells experience Sec-translocon capacity problems they increase their SecA levels (Nakatogawa and Ito, 2001). The increased LepB accumulation levels indicate that cells have a higher capacity to process secretory proteins. The increased YidC accumulation levels indicate that cells have an increased capacity to support the biogenesis of membrane proteins thereby relieving the Sec-translocon capacity (Figure 2C). It has been shown before that protein translocation stress at the Sec-translocon levels can lead to increased YidC accumulation levels (Baumgarten et al., 2018). The increased YidC levels could compensate for insufficient Sec-translocon capacity.

Taken together, our observations indicate that enhancing the periplasmic production of hGH leads to the enhanced protein translocation capacity of the cell and there seems to be a correlation between periplasmic hGH production yields and the increase in accumulation levels of components involved in protein translocation. When hGH is produced in the periplasm using Hbp_sp_ at increasing concentrations of rhamnose not only periplasmic hGH production yields gradually increase but also SecA, LepB, and YidC levels do, until hGH production in the periplasm cannot be further enhanced (Supplementary Figure 1). This supports the idea that there is a correlation between enhancing periplasmic hGH production yields using the rhamnose promoter setup and the increase of the protein translocation capacity of the cell.

### Does the Protein Translocation Machinery Adapt in Response to Enhancing Periplasmic hGH Production?

**Figure 3:**
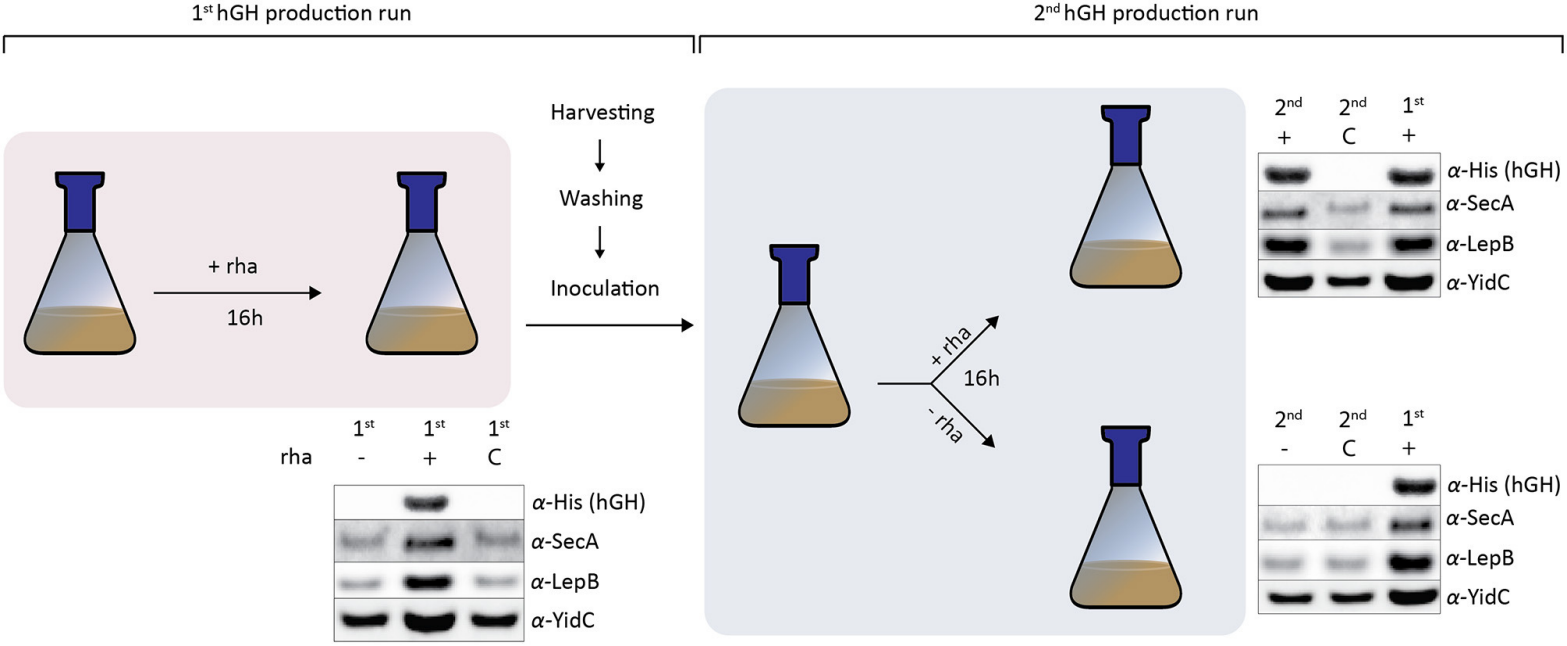
Cells increase their SecA, LepB, and YidC levels in response to enhanced periplasmic hGH production. In the first hGH production run using Hbp_sp_, 16 h after induction with rhamnose, hGH, SecA, LepB, and YidC accumulation levels were monitored by means of SDS-PAGE/immuno-blotting. Cells with a pRha-*hbp*_*sp*_*hghhis*_6_ expression vector to which no inducer (–) was added and cells with an empty expression vector (C) cultured in the presence of inducer were used as controls. The accumulation levels of hGH, SecA, LepB, and YidC were monitored using antisera against the C-terminal His_6_-tag of hGH, SecA, LepB, and YidC. For SecA, LepB, and YidC a secondary fluorescently labeled antibody was used for visualization. Equal amounts of cells based on A_600_ measurements were loaded. To set up the second hGH production run, cells producing hGH and control cells with an empty expression vector were extensively washed in medium without rhamnose and used to inoculate fresh cultures. When the culture had reached an A_600_ of ~0.5 the culture was split. To one half of the culture rhamnose was added and to the other half no rhamnose was added. 16 h after the addition of rhamnose in the second hGH production run, cells were harvested and hGH, SecA, Lep, and YidC levels were monitored as described above. Cells producing hGH in the first hGH production run were included as a reference.

To study if the nature of the increased SecA, LepB, and YidC accumulation levels upon enhanced hGH production in the periplasm was permanent or reversible, cells producing hGH in the periplasm using Hbp_sp_ were used to set up cultures with and without inducer (Figure 3). Cells with an empty expression vector cultured in the presence of inducer were used as a control. In the first hGH production run 16 h after induction, hGH, SecA, LepB, and YidC accumulation levels were monitored by means of immuno-blotting (Figure 3). As expected, the production of hGH led to increased SecA, LepB, and YidC accumulation levels.

The cells producing hGH and control cells were extensively washed in medium without rhamnose and used to inoculate a fresh culture for the second hGH production run. When the culture had reached the density for induction the culture was split. To one half of the culture 50 μM rhamnose was added and to the other half no rhamnose was added. Sixteen hours after the addition of rhamnose, cells were harvested and hGH, SecA, LepB, and YidC levels were monitored by means of immuno-blotting. The addition of inducer led to efficient periplasmic hGH production and increased SecA, LepB, and YidC accumulation levels. Not adding inducer resulted in no hGH production and SecA, LepB, and YidC accumulation levels that were similar to the ones in the control cells.

Taken together, cells producing hGH can be used as starting material to set up a new culture for the production of hGH, whereas when no rhamnose was added no hGH was produced and SecA, LepB, and YidC accumulation levels went back to levels of cells not producing periplasmic hGH. This indicates that enhancing periplasmic production of hGH using the rhamnose promoter-based production setup leads to the adaptation of its protein translocation machinery. When the same approach as outlined in Figure 3 was used to produce the single chain antibody fragment BL1 in the periplasm, the effects on the accumulation levels of SecA, LepB, and YidC were the same as the ones observed for the production of hGH in the periplasm (Supplementary Figure 2), which indicates that these effects are not specific for hGH production.

## Concluding Remarks

Here, we have shown that enhancing the production of a recombinant protein in the periplasm of *E. coli* by rhamnose promoter-based production rate screening can lead to increased accumulation levels of at least three key players in protein translocation, SecA, LepB, and YidC. It is conceivable that the accumulation levels of more key players involved in protein translocation also go up. Interestingly, the increase of the SecA, LepB, and YidC accumulation levels is reversible, which indicates that *E. coli* adapts for enhanced periplasmic recombinant protein production through regulatory mechanisms rather than through the accumulation of mutations. Previously, it has been shown that when the strong T7 promoter is used for recombinant protein production, stress caused by the production of a recombinant protein can lead to the accumulation of mutations that alleviate production stress while at the same time they enable enhanced production yields (Kwon et al., 2015; Schlegel et al., 2015). Thus, the promoter system governing the expression of the gene encoding a recombinant protein seems to play a role in how *E. coli* deals with recombinant protein production stress.

How does *E. coli* adapt for enhanced periplasmic recombinant protein production when using the tunable rhamnose promoter-based setup? It is tempting to speculate that enhancing production yields by protein production rate screening using the tunable rhamnose promoter-based setup leads to mild protein secretion stress to which cells can adapt by increasing their protein translocation capacity, rather than as previously thought the direct harmonization of secretory recombinant protein production rates with the Sec-translocon capacity (Schlegel et al., 2013). It has been shown that in *E. coli* the SecA-dependent secretion monitor SecM is involved in secretion-responsive control of SecA translation; i.e., inefficient translocation of SecM to the periplasm *via* the Sec-translocon results in the increased synthesis of SecA in order to relieve the protein translocation stress (Nakatogawa and Ito, 2001; Murakami et al., 2004; Nakatogawa et al., 2004). Thus, insufficient Sec-translocon capacity upon secretory recombinant protein production screening explains the increased SecA accumulation levels. The mechanism(s) behind the upregulation of LepB and YidC remains to be elucidated. Interestingly, when the PhoA_sp_ is used for the periplasmic production of hGH, periplasmic protein production yields are much lower than for the other three signal peptides used (Karyolaimos et al., 2019). When using the PhoA_sp_ only the accumulation levels of SecA and LepB go up and their increase is by far not as high as for the other signal peptides. YidC accumulation levels are not affected when PhoA_sp_ is used. This indicates that when using PhoA_sp_ to produce periplasmic hGH, increasing SecA and LepB levels is sufficient to deal with the secretory recombinant protein production stress. At any rate, our study indicates that *E. coli* has, besides the secretion monitor SecM, also other mechanisms enabling it to adapt its protein translocation capacity to its protein translocation needs. It is actually very well-conceivable that *E. coli* encounters in its natural habitat(s) conditions that require it to adapt its protein translocation machinery.

Taken together, it appears that we have exploited *E. coli*'s ability to modulate its protein translocation machinery capacity to enhance the production of recombinant proteins in the periplasm. Cracking the mechanism(s) behind *E. coli*'s ability to modulate its protein translocation capacity may provide the basis for engineering the next generation of strains for periplasmic recombinant protein production.

## Data Availability Statement

The datasets generated for this study can be found in the mass spectrometry proteomics data have been deposited to the ProteomeXchange Consortium (http://proteomecentral.proteomexchange.org) via the PRIDE partner repository with the dataset identifier PXD013168 (username: reviewer89655@ebi.ac.uk, password: myvMo3bU).

## Author Contributions

AK, KD, MA-V, AM, and J-WG conceived and designed the experiments. AK, KD, MA-V, AM, and RE performed the experiments. AK, KD, MA-V, AM, RE, SS, DB, KR, and J-WG analyzed the data. AK and J-WG wrote the paper and all others critically read the paper.

### Conflict of Interest

The authors declare that the research was conducted in the absence of any commercial or financial relationships that could be construed as a potential conflict of interest.
