# Supplementary material for: Escherichia coli Can Adapt Its Protein Translocation Machinery for Enhanced Periplasmic Recombinant Protein Production
Source: Front Bioeng Biotechnol. 2020 Jan 29;7:465. doi: 10.3389/fbioe.2019.00465 (PMC7000420; doi:10.3389/fbioe.2019.00465)
Supplement: Supplementary file 1 [file Data_Sheet_1.pdf]

Supplementary Information

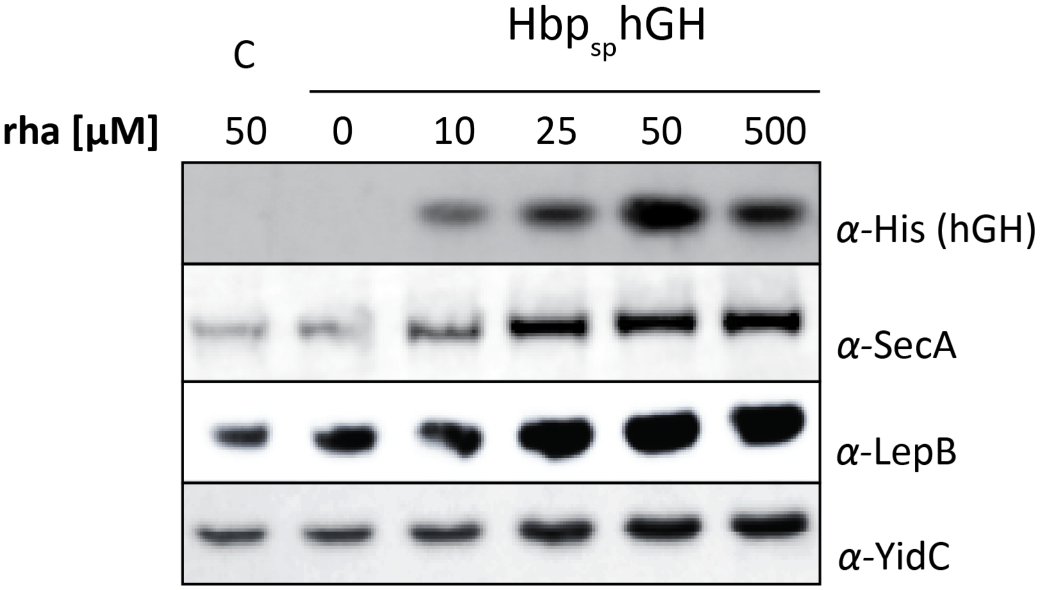

Supplementary figure 1. SecA, LepB and YidC accumulation levels increase gradually with increasing hGH production levels in the periplasm. hGH N-terminally fused to Hbp$_{sp}$ was produced in *E. coliΔrha* at increasing concentrations of rhamnose (0, 10, 25, 50 and 500 µM). It should be noted that 50 µM rhamnose is the optimal concentration of rhamnose for the periplasmic production of hGH when using Hbp$_{sp}$. *E. coliΔrha* harbouring an empty expression vector cultured in the presence of 50 µM rhamnose was used as a control. Cells were harvested 16 hours after induction of target gene expression with rhamnose and equal amounts of cells were analyzed by SDS-PAGE followed by immuno-blotting to monitor hGH production levels as well as SecA, LepB and YidC accumulation levels as described in the Materials and Methods section.

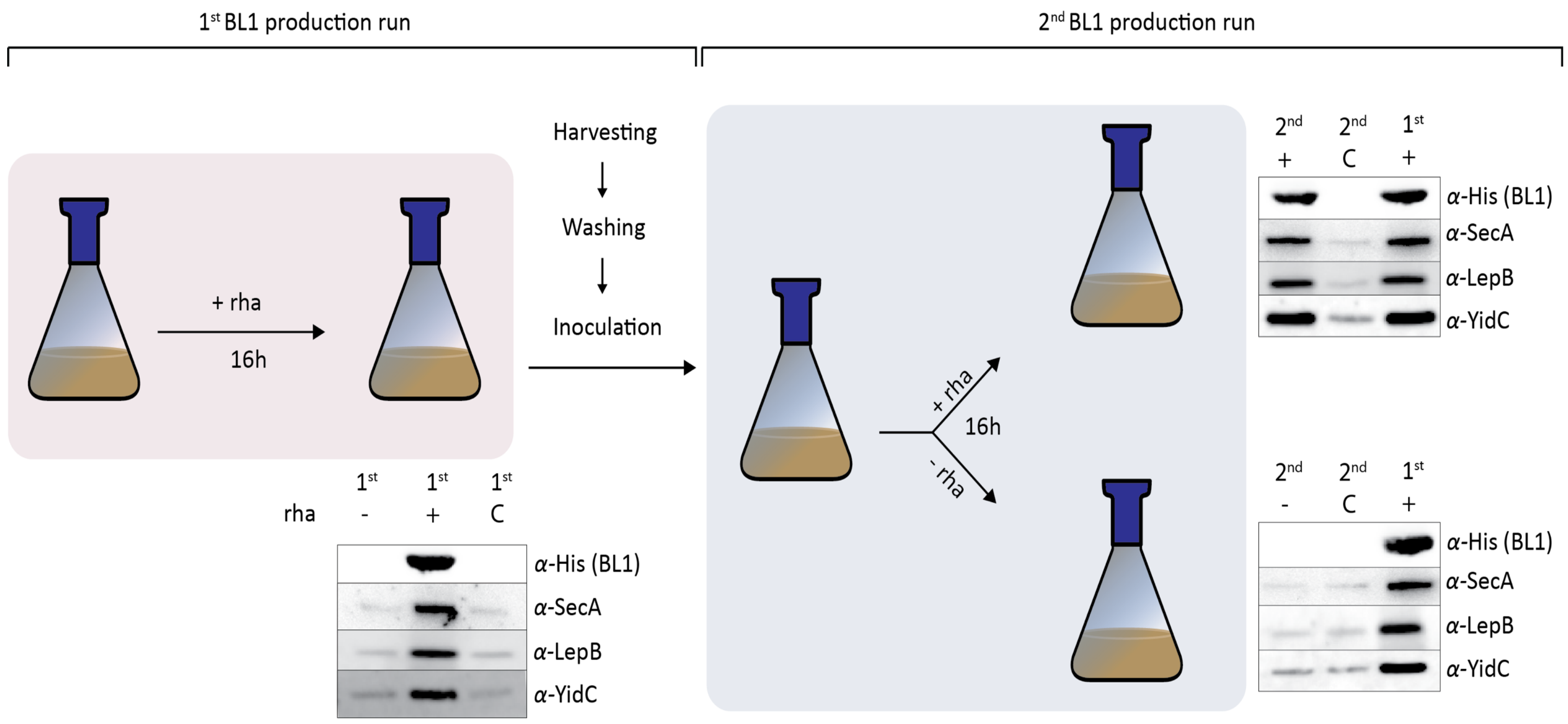

1st BL1 production run
2nd BL1 production run
+ rha
16h
Harvesting
Washing
Inoculation
+ rha
- rha
16h
rha
1st 1st 1st
- + C
α-His (BL1)
α-SecA
α-LepB
α-YidC
2nd 2nd 1st
+ C +
α-His (BL1)
α-SecA
α-LepB
α-YidC
2nd 2nd 1st
- C +
α-His (BL1)
α-SecA
α-LepB
α-YidC

Supplementary figure 2. Cells increase their SecA, LepB and YidC levels in response to enhanced periplasmic scFv BL1 production. In the first scFv BL1 production run using $OmpA_{sp}$, 16 hours after induction with rhamnose, scFv BL1, SecA, LepB, and YidC accumulation levels were monitored by means of SDS-PAGE/immuno-blotting. Cells with a pRha-*ompA*$_{sp}$*bl1his*$_6$ expression vector to which no inducer (-) was added and cells with an empty expression vector (C) cultured in the presence of inducer were used as controls. The accumulation levels of scFv BL1, SecA, LepB and YidC were monitored using antisera against the C-terminal His_6-tag of scFv BL1, SecA, LepB and YidC. For SecA, LepB and YidC a secondary fluorescently labelled antibody was used for visualization. Equal amounts of cells based on A_{600} measurements were loaded. To set up the second scFv BL1 production run, cells producing hGH and control cells with an empty expression vector were extensively washed in medium without rhamnose and used to inoculate fresh cultures. When the culture had reached an A_{600} of ~0.5 the culture was split. To one half of the culture rhamnose was added and to the other half no rhamnose was added. 16 hours after the addition of rhamnose in the second scFv BL1 production run, cells were harvested and scFv BL1, SecA, Lep and YidC levels were monitored as described above. Cells producing scFv BL1 in the first hGH production run were included as a reference.
